# Adherence to prehospital thoracostomy practice guidelines for traumatic cardiac arrest: A retrospective study

**DOI:** 10.1016/j.resplu.2025.100870

**Published:** 2025-01-16

**Authors:** Nicolas Beysard, Tara Agudo, Peter Serfozo, Tobias Zingg, Perrine Truong, Roland Albrecht, Vincent Darioli, Mathieu Pasquier

**Affiliations:** a Department of Emergency Medicine, Lausanne University Hospital and University of Lausanne, Rue du Bugnon 46, 1011 Lausanne, Switzerland; b Faculty of Biology and Medicine, University of Lausanne, Rue du Bugnon 21, 1011 Lausanne, Switzerland; c Department of Visceral Surgery, Lausanne University Hospital, Rue du Bugnon 46, 1011 Lausanne, Switzerland; d Swiss Air-Rescue (Rega), Zurich, Switzerland

**Keywords:** Traumatic cardiac arrest, Thoracostomy, Prehospital, Adherence, Guidelines

## Abstract

**Objectives:**

The management of traumatic cardiac arrest (TCA) focuses on the immediate treatment of reversible causes, including bilateral thoracostomy. In our prehospital emergency service, bilateral thoracostomy has been recommended since 2012 for the management of TCA. We sought to analyse the prehospital management and clinical course of patients with TCA, focusing on changes over time in the use of thoracostomy.

**Methods:**

In this single-centre retrospective observational study, we included patients with TCA managed by physicians of the prehospital service of Lausanne University Hospital from 2012 to 2024. The primary outcome was the annual rate of bilateral thoracostomy. Secondary outcomes included the rate of additional on-site measures, such as pelvic binder placement and airway management, and follow-up at 48 h.

**Results:**

Among 3206 cardiac arrests during the study period, 473 (15%) were TCAs. Among the 247 patients with resuscitation attempts, thoracostomy was judged as indicated in 223 (90%) and performed in 148 (66%). Twenty-seven (18%) patients who had a thoracostomy were alive on arrival at hospital, with 9 (6.1%) still alive at 48 h. The mean annual proportion of patients in whom a thoracostomy was performed was 68% (range 0–100%) and increased significantly over the years (*p* < 0.001).

**Conclusions:**

The annual rate of thoracostomy in TCA patients increased significantly in the period 2012 to 2024. Larger studies are required to determine the impact of thoracostomy on survival.

## Introduction

According to the World Health Organization, 4.4 million injury-related deaths occur every year.^1^ Worldwide, road traffic deaths represent the eighth leading cause of death in people of all ages and the first cause of death in children and young adults 5–29 years of age.^2^ For many years, traumatic cardiac arrest (TCA) was considered to have an extremely poor prognosis and trauma resuscitation to be nearly futile.^3^ Authors of more recent studies, however, have observed a survival rate of up to 7.5%.^4^, ^5^, ^6^ Moreover, some deaths were reported to be potentially preventable, justifying the development of specific management guidelines.^4^, ^7^

Prior to 2005, prehospital care guidelines were unspecific, recommending rapid transport to the nearest trauma centre. In 2005, recommendations suggested performing essential lifesaving interventions on scene before rapid transfer if the patient has signs of life.^8^ Since 2005, on-scene resuscitative thoracotomy was to be considered in TCA with penetrating chest injury. In 2010, the main change was the recommendation that cardiopulmonary resuscitation not delay any life-saving measures.^9^ This recommendation was reinforced in 2015 with the statement that chest compressions should take lower priority than the immediate treatment of reversible causes, with the addition of bilateral thoracostomy and pericardiocentesis to the guidelines.^10^ Since 2012, our service’s internal recommendations suggest performing bilateral thoracostomy in TCA when thoracic trauma cannot be excluded. Even though indication for performing bilateral thoracostomy has remain the same since 2012, the timing of performance has evolved. Bilateral thoracostomy was recommended in previously intubated patients initially, as since 2014, it was proposed before advanced airway management.

Adherence to resuscitation guidelines in TCA is known to be poor,^11^ even though adherence to guidelines tends to improve survival.^12^, ^13^ We aimed to analyse the prehospital management and clinical course of patients with TCA, focusing on adherence to medical guidelines, especially regarding bilateral thoracostomy.

## Material and methods

### Study design

This is a single-centre retrospective observational study including patients suffering from out of hospital traumatic cardiac arrest managed by physicians of the prehospital service of the Lausanne University Hospital emergency department (ED).

### Setting

The prehospital emergency physicians of the Lausanne University Hospital staff a 24/24, 7/7 helicopter emergency medical service (HEMS) and a ground emergency medical service (GEMS). The HEMS intervenes in a 5,600 km^2^ area that includes about 1,400,000 inhabitants. The GEMS serves an urban (150 km^2^) and suburban (400 km^2^) area with a population of about 450,000 inhabitants. Patient information is saved in a digital medical record. This includes general and medical information, as well as any performed interventions, such as intubation, thoracostomy, pericardiocentesis, pelvic binder application, intravenous (IV) or intraosseous (IO) insertion, and non-invasive ventilation. The medical chart also includes a systematic 48-hour follow-up for every patient who has not died on-site.

We included patients managed by physicians of the prehospital service of the Lausanne University Hospital emergency department (ED) from January 1, 2012, to June 30, 2024. The study was approved by our regional ethical committee (CER-VD 2022-00992).

We included the following data: year of intervention, age, sex, means of transport (HEMS or GEMS), type (penetrating or blunt) and mechanism of trauma, presumed cause of cardiac arrest by physician on-site, and whether resuscitation was attempted or not. We analysed whether the following medical procedures were performed on patients with resuscitation attempts: external wound pressure, pressure dressings, tourniquet application, airway management (defined as endotracheal intubation or bag-mask ventilation), application of pelvic binder, IV or IO fluid infusion, needle pneumothorax decompression, pericardiocentesis, and bilateral thoracostomy. A 48-hour follow-up was available for patients brought to the hospital. The following patient outcomes were recorded: 48-hour survival status, time and site of death if relevant, injury severity score, abbreviated injury scale category with the most points, final recorded cardiac rhythm (at death or return of spontaneous circulation (ROSC)), site of death if relevant, and immediate treatments after admission to the ED (surgery within 0–4 h, chest tube). Specific data for patients in whom thoracostomies and pericardiocentesis were performed included the following: presence of a suspected or obvious thoracic trauma, whether the patient was already intubated at the time of thoracostomy, cardiac rhythm before and after thoracostomy, and technical success of thoracostomy (defined as the success of reaching the intrathoracic cavity according to the digital exploration by the prehospital physician).

Our primary outcome was the proportion of patients for whom resuscitation was initiated and with an indication for bilateral thoracostomy according to guidelines, and in whom bilateral thoracostomy was actually performed. Patients in whom resuscitation was not attempted were assumed to be correctly handled by default. For patients with a resuscitation attempt, we considered bilateral thoracostomy as indicated if the diagnostic category as coded by the emergency physician included thoracic trauma. For the remaining cases, the prehospital chart was searched for information by one of the authors (NB) to decide whether bilateral thoracostomy were indicated or not. If there was a possible thoracic trauma, NB classified the case as “bilateral thoracostomy indicated”. In case of doubt, a consensus was reached after discussion with another author (MP). NB and MP are senior emergency physicians with extensive experience in pre-hospital medicine. When bilateral thoracostomy was considered indicated, we considered it appropriate if only a unilateral thoracostomy was performed in the case where the patient had a ROSC before the second thoracostomy was performed and if ROSC was sustained.

Secondary outcomes included performed medical procedures (tourniquet placement, pressure dressing, airway management, oxygenation, needle pneumothorax decompression, pericardiocentesis, IV or IO fluid infusion, pelvic binder application).

### Statistical analysis

The data were exported from RedCap to Stata version 18 (Stata Corporation, College Station, TX, USA). Continuous variables were expressed as mean and standard deviation (SD) or as median and interquartile range, depending on the data distribution. Categorical variables were reported as absolute numbers and percentages. Continuous variables were compared by using Student's *t*-test when normally distributed and by using the Wilcoxon-Mann-Whitney test when non-normally distributed. We used Pearson’s chi-square test and Fisher's exact test to compare categorical variables. We used Spearman’s correlation co-efficient to explore the relationship between annual 48-hour survival rates. As only 6 months of the year in 2024 were included in the present study, figures were annualized where relevant (e.g. for calculating annual rates) to allow for comparison. A two-tailed p-value of < 0.05 was considered statistically significant.

## Results

**Fig. 1:**
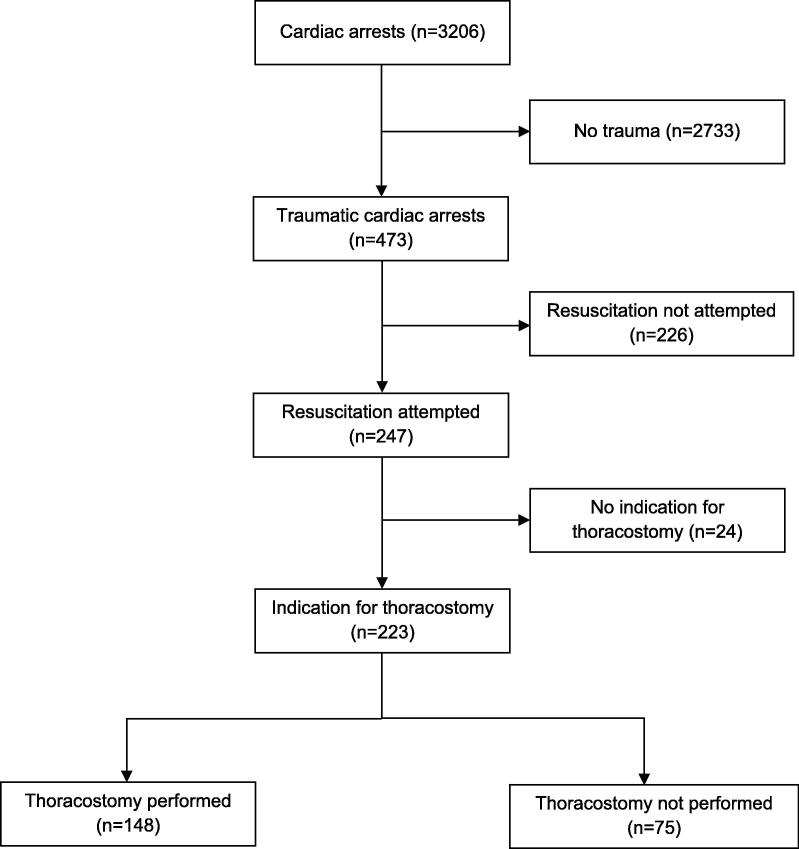
Flow chart of study patients. Patients with traumatic cardiac arrest (TCA) managed by prehospital emergency physicians of Lausanne University Hospital, Switzerland, 2012–2024.

Among 3206 cardiac arrests during the study period, 473 (14.8%) were TCAs (Fig. 1). The mean annual number of TCAs was 38 (SD 7, range 30–53) and remained stable over the years (*p* = 0.59). Resuscitation was attempted in 247 of 473 (52.2%) patients. The mean annual proportion of patients with resuscitation attempts was 53% (SD 8, range 38–65%) and remained stable over the years (*p* = 0.48). The general characteristics of the study population are presented in Table 1.

**Table 1:** Characteristics of patients with traumatic cardiac arrest managed by prehospital emergency physicians of Lausanne University Hospital, Switzerland, 2012–2024 (*n* = 473).

	**Overall (*n* = 473)**	**Resuscitation attempted (*n* = 247)**	**Resuscitation not attempted (*n* = 226)**	**P-value**
**Age, years, mean (SD)**	47 (±21)	45 (±21)	50 (±21)	<0.035
**Male, n (%)**	369 (78.0)	200 (80.9)	169 (74.8)	0.662
**Means of transport, n (%)**				0.061
GEMS	234 (49.5)	112 (45.3)	122 (53.9)	
HEMS	239 (50.5)	135 (54.7)	104 (46.1)	
**Type of trauma, n (%)**				0.947
Blunt	416 (87.9)	217 (87.9)	199 (88.1)	
Penetrating	57 (12.1)	30 (12.1)	27 (11.9)	
**Mechanism of trauma, n (%)**				<0.001
Fall of more than 3 m	179 (37.8)	68 (27.5)	111 (49.1)	
Pedestrian	71 (15.1)	23 (9.3)	48 (21.2)	
≥4-wheeled vehicle accident	64 (13.5)	42 (17.0)	22 (9.8)	
2-wheeled vehicle accident	61 (12.9)	56 (22.6)	5 (2.2)	
Firearm	36 (7.6)	12 (4.9)	24 (10.6)	
Stabbing	13 (2.7)	12 (4.9)	1 (0.4)	
Other	49 (10.4)	34 (13.8)	15 (6.7)	
**On-site presumed cause of cardiac arrest, n (%)**				<0.001
Polytrauma	244 (51.6)	129 (52.2)	115 (510.9)	
Severe traumatic brain injury	115 (24.3)	52 (21.1)	63 (27.8)	
Thoracic trauma	32 (6.8)	25 (10.1)	7 (3.1)	
Exsanguination	19 (4.0)	17 (6.9)	2 (0.9)	
Other/not specified	63 (13.3)	24 (9.7)	39 (17.3)	

GEMS: ground emergency medical service; HEMS: helicopter emergency medical service.

**Fig. 2:**
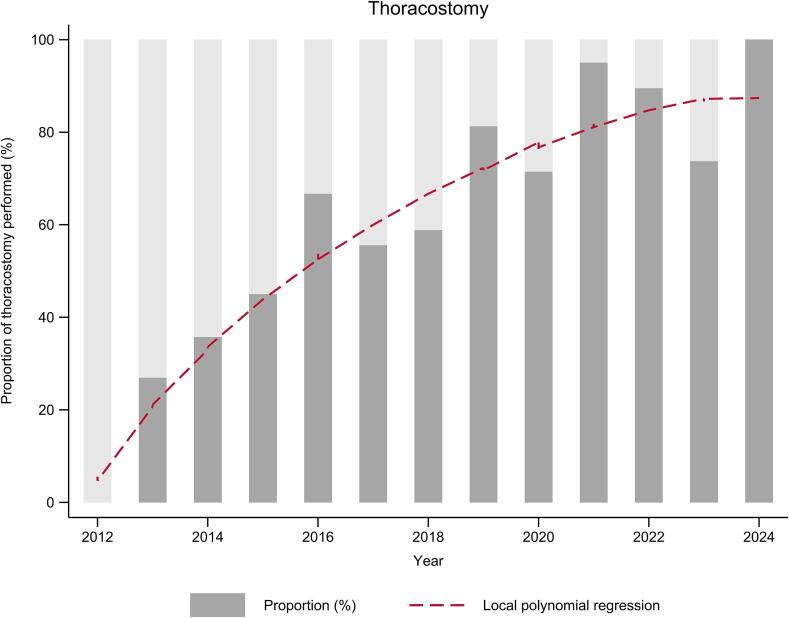
Annual proportion of patients in whom resuscitation was attempted and with an indication for thoracostomy, and in whom a thoracostomy and/or a pelvic binder was applied (*n* = 223), with traumatic cardiac arrest managed by the prehospital emergency physicians of the Lausanne University Hospital, Switzerland, 2012–2024.

Among the 247 patients with resuscitation attempts, thoracostomy was judged as not indicated in 24 (9.7%). The presumed cause of TCA was isolated severe traumatic brain injury in 19 (79.2%) and exsanguination in 4 (16.7%) and polytrauma with a ROSC soon after the physician's arrival on site in 1 (4.1%). Three patients (13%) were alive at 48 h. Thoracostomy was judged as indicated in 223 (90.3%) of the 247 patients with resuscitation attempts and performed in 148 (66.4%). The mean annual proportion of patients in whom an indicated thoracostomy was performed was 68% (range 0–100%) and increased significantly over the years (*p* < 0.001) (Fig. 2). The characteristics of the patients with an indication for thoracostomy, depending on whether it was performed or not, are presented in Table 2.

**Table 2:** Characteristics of patients in whom resuscitation was attempted and with a presumed indication for bilateral thoracostomy, depending on whether it was performed or not (*n* = 223), with traumatic cardiac arrest (TCA) managed by the prehospital emergency physicians of the Lausanne University Hospital, Switzerland, 2012–2024.

	**Overall (*n* = 223)**	**Thoracostomy performed (*n* = 148)**	**Thoracostomy not performed (*n* = 75)**	**P-value**
**ERC guidelines valid at that time, n (%)**				<0.001
2010	68 (30.5)	21 (14.2)	47 (62.6)	
2015	98 (43.9)	72 (48.6)	26 (34.7)	
2021	57 (25.6)	55 (37.2)	2 (2.7)	
**Age, years, mean (SD)**	45 (±21)	45 (±21)	45 (±22)	0.916
**Male, n (%)**	179 (80.3)	120 (81.1)	59 (78.7)	0.669
**Means of transport, n (%)**				0.398
GEMS	104 (46.6)	72 (48.6)	32 (42.7)	
HEMS	119 (53.4)	76 (51.4)	43 (57.3)	
**Type of trauma, n (%)**				0.757
Blunt	204 (91.5)	136 (91.9)	68 (90.7)	
Penetrating	19 (8.5)	12 (8.1)	7 (9.3)	
**On-site presumed cause of cardiac arrest, n (%)**				0.415
Polytrauma	128 (59.5)	89 (62.2)	39 (54.2)	
Severe trauma brain injury	33 (15.4)	20 (14.0)	13 (18.1)	
Thoracic trauma	25 (11.6)	18 (12.6)	7 (9.7)	
Exsanguination	13 (6.1)	6 (4.2)	7 (9.7)	
Other/not specified	16 (7.4)	10 (7.0)	6 (8.3)	
**Medical management, n (%)**				
Airway management	219 (98.2)	146 (98.6)	73 (97.3)	0.604
Oxygenation	209 (93.7)	138 (93.2)	71 (94.7)	0.778
IV or IO fluid infusion	198 (88.7)	135 (91.2)	63 (84.0)	0.119
Pelvic binder	123 (55.2)	95 (64.2)	28 (37.3)	<0.001
Pneumothorax needle decompression	40 (17.9)	24 (16.2)	16 (21.3)	0.360
Pericardiocentesis^a^	22 (9.9)	21 (14.2)	1 (1.3)	0.001
Pressure dressings	12 (5.4)	9 (6.1)	3 (4.1)	0.755
External wound pressure	9 (4.1)	6 (4.2)	3 (4.0)	1
Tourniquet	5 (2.2)	3 (2.0)	2 (2.7)	1
**Outcomes**				
ISS, median (IQR)	75 (43–75)	75 (47–75)	75 (41–75)	0.941
AIS category with most points, n (%)				0.370
Head/neck	90 (56.9)	63 (54.8)	27 (62.7)	
Thorax	46 (29.1)	36 (31.3)	10 (23.3)	
Abdomen	8 (5.1)	4 (3.5)	4 (9.3)	
Extremities/pelvis	11 (7.0)	9 (7.8)	2 (4.7)	
Face	3 (1.9)	3 (2.6)	0 (0)	
48-hour survival^b^, n (%)	11 (4.9)	9 (6.1)	2 (2.7)	0.342
**Site of death, n (%)**				<0.002
On-site	166 (74.4)	121 (81.7)	45 (60.0)	
In hospital	43 (19.3)	18 (12.2)	25 (33.3)	
**Immediate treatment after hospital admission, n (%)**^c^				0.381
Chest tube insertion	13 (24.1)	4 (14.8)	9 (33.3)	
Surgery within 0–4 h	15 (27.8)	9 (33.3)	6 (22.2)	

AIS: abbreviated injury scale; ERC: European Resuscitation Council; GEMS: ground emergency medical service; HEMS: helicopter emergency medical service; IO: intraosseous; IQR: interquartile range; ISS: injury severity score; IV: intravenous.

a 19 (86.4%) blunt trauma of 22, 3 (13.6%) penetrating of 22 penetrating. Return of spontaneous circulation was described immediately after pericardiocentesis in 2 patients (9.1%), and 1 was alive at 48 h (4.5%).

b p remained non-significant (*p* = 0.73) when analysing the survival after having included the 16 cases in which a pneumothorax needle decompression was performed in the “thoracostomy performed” group.

c Excluding patients dead on site (*n* = 54 for overall (24.2%), *n* = 27 for thoracostomy performed (18.2%) and *n* = 27 for thoracostomy not performed (36.0%).

Cardiac rhythms before thoracostomy were documented in 144 cases (97.3%) or in 140 cases (94.9%) after thoracostomy. Cardiac rhythms before thoracostomy were asystole (96 of 144 = 66.7%), pulseless electrical activity (PEA) (42 of 144 = 29.2%), and ventricular fibrillation or pulseless ventricular tachycardia (VF-pVT) (5 of 144 = 3.5%). Cardiac rhythms after thoracostomy were asystole (76 of 140 = 54.3%), PEA (46 of 140 = 32.9%) and VF-pVT (7 of 140 = 5%). Among the 148 thoracostomies performed, prehospital emergency physicians reported a procedural success in 134 cases (90.5%), with only one case documented as a failure (0.7%) and 13 cases in which information was not available (8.8%). Twenty-seven (18.2%) of the patients who had a thoracostomy were transported alive to hospital, and nine (6.1%) were alive at 48 h. Thoracostomy was performed before intubation in 92 (62.2%) of the 148 patients for whom this information was available.

Of the 473 patients, 15 (3.2%) were alive at 48 h, including four who had no indication for thoracostomy, representing 5.7% of the 247 patients with resuscitation attempts. The Spearman correlation test suggested an increase in survival over the years, without reaching statistical significance (rho = 0.51, *p* = 0.075).

## Discussion

In this single-centre retrospective observational study the mean annual number of TCA resuscitation attempts has remained stable while the annual proportion of TCA patients with resuscitation attempts in whom a thoracostomy was performed increased significantly over the years. It was not difference in survival among patients with or without thoracostomy.

Identification of potentially reversible causes is crucial in the management of TCA. Bilateral thoracostomy is increasingly recognized as a fundamental early rescue measure, especially in the prehospital environment.^14^, ^15^, ^16^, ^17^ The progressive implementation of TCA-specific guidelines led to a rise in the delivery of systematic pleural decompressions.^6^, ^13^ Bilateral thoracostomy was introduced in 2012 in the Lausanne University prehospital emergency service for the management of TCA in cases where thoracic trauma could not be clinically excluded. These guidelines were based on studies suggesting that one of the potentially reversible causes of TCA was tension pneumothorax and that thoracostomy should be considered instead of chest needle decompression.^7^, ^18^, ^19^, ^20^, ^21^ Education and basic training were provided to all physicians in order to be familiar with performing bilateral thoracostomy. Initially, bilateral thoracostomy was recommended only in previously intubated patients. Since 2014, we have suggested performing bilateral thoracostomy before advanced airway management and minimizing the urgency for chest compressions, as external cardiac massage is ineffective in the presence of tension pneumothorax until the elevated intrathoracic pressure is released.^4^ In 2015, prehospital emergency physicians were specifically trained to perform bilateral thoracostomy with dedicated scenario and skills training on high-fidelity manikin in order to perform as quickly as possible the bilateral thoracostomy, regardless of the cause and the initial rhythm.^22^ Although the mean annual number of TCA resuscitation attempts has remained stable over the years, the percentage of thoracostomies performed on-site has significantly increased. This rise correlates with the progressive incorporation of thoracostomy in the management of TCA. The impact of these changes is evident when comparing the percentages of thoracostomies performed under different European Resuscitation Council guidelines: more precise guidelines, repetitive training, and teaching over the years improves adherence.^12^, ^23^

The frequency of thoracostomy in patients with TCA increased significantly over the study period, with an annual proportion ranging from 0% in 2012 to 100% in 2023. Savary et al. also reported a marked rise in bilateral thoracostomy following the implementation of specific local TCA recommendations in their emergency network in France in 2008.^13^ They described an augmentation from 5% in the pre-guideline period to 100% in the post-guideline period. In a 2021 retrospective study from the Netherlands, Houwen et al. reported a thoracic decompression rate, including needle and finger thoracostomy, of 58.7% between 2014 and 2018.^15^ A retrospective study by Avest et al. showed that thoracostomy was the most prevalent prehospital intervention delivered to TCA patients (84%).^24^ We cannot exclude that some patients for whom a bilateral thoracostomy was indicated did not received it because their injuries were deemed too severe or because a ROSC was obtained before performing it, leading to a potential resuscitation time bias.^25^

To date, despite increasing implementation and adherence to TCA guidelines, no better survival outcomes have been observed.^6^, ^13^, ^26^ In our study, of the 473 patients, 15 (3.2%) survived. This low survival rate allows no conclusive assessment of the impact of thoracostomy on survival.

Chest tubes were inserted on arrival at the ED in nine (33.3%) patients when thoracostomies were not performed on-site, and only two (21.3%) had needle decompression performed. This result highlights the importance of following the guidelines, as the on-site clinically estimated cause of TCA seems to be insufficient to exclude a pneumothorax.^7^

Point-of-care ultrasonography (POCUS) is an emerging diagnostic tool in prehospital care.^27^, ^28^ Although several studies confirmed its feasibility in cardiac arrest, there are still several concerns such as prolonged pauses in chest compressions.^29^, ^30^ We hypothesize that identification of reversible causes of TCA by systematic focused ultrasonography, using, for example, an eFAST protocol to look for pneumothorax and pericardial, pleural, or abdominal free fluid, may help determine the order of interventions (e.g. pericardiocentesis before thoracostomy in the case of pericardial tamponade).

Future studies should focus on determining the impact of TCA-specific prehospital measures, such as bilateral thoracostomy on survival, based on larger cohort. The development and assessment of standardized TCA-specific POCUS protocols should also be explored.

## Limitations

Our study has several limitations. Because of its retrospective design, data completeness relies on accurate recordkeeping, which is particularly challenging in the prehospital environment. We assumed that the on-site physician decision not to initiate resuscitation was correct, which could have caused us to miss cases for which there was no reason to withhold resuscitation. The decision whether bilateral thoracostomy were indicated or not was not blinded, and doubtful cases were resolved by consensus. This was the case for only 3 patients. It's not much and shouldn't skew our results. We have identified a lower rate of penetrating injuries compared with worldwide prevalence, which clearly limits generalizability. The small number of patients does not allow a conclusive assessment of the impact of thoracostomies on survival. Finally, survival to discharge and neurological outcomes of survivors were not assessed, limiting interpretability.

## Conclusions

In this single-centre retrospective observational study, adherence to TCA guidelines, in particular bilateral thoracostomy improved significantly over the period 2012 to 2024. The impact of these measures on survival remains unknown. Future studies dedicated specifically to this question are needed.

## CRediT authorship contribution statement

**Nicolas Beysard:** Writing – original draft, Software, Methodology, Investigation, Formal analysis, Data curation, Conceptualization. **Tara Agudo:** Writing – original draft, Methodology, Investigation, Conceptualization. **Peter Serfozo:** Writing – original draft, Investigation. **Tobias Zingg:** Writing – review & editing. **Perrine Truong:** Writing – original draft, Investigation. **Roland Albrecht:** Writing – review & editing. **Vincent Darioli:** Writing – review & editing. **Mathieu Pasquier:** Writing – original draft, Validation, Supervision, Methodology, Formal analysis, Conceptualization.

## Funding

This research did not receive any specific grant from funding agencies in the public, commercial, or not-for-profit sectors.

## Declaration of competing interest

The authors declare that they have no known competing financial interests or personal relationships that could have appeared to influence the work reported in this paper.
